# Low-Level Viremia in People Living with HIV: A Retrospective Cohort Study

**DOI:** 10.3390/v18060611

**Published:** 2026-05-27

**Authors:** Uğur Önal, Egemen Özdemir, Şeyma Öncül, Hazel Öztürk Belik, Esra Kazak, Yasemin Heper, Harun Ağca, Emel Yılmaz, Halis Akalın

**Affiliations:** 1 Department of Infectious Diseases and Clinical Microbiology, Faculty of Medicine, Bursa Uludağ University, Bursa 16059, Türkiye; egemenozdemir@uludag.edu.tr (E.Ö.); seymaoncul@gmail.com (Ş.Ö.); hazelozturk@uludag.edu.tr (H.Ö.B.); eskazak@uludag.edu.tr (E.K.); yheper@uludag.edu.tr (Y.H.); emelyilmaz@uludag.edu.tr (E.Y.); halis@uludag.edu.tr (H.A.); 2 Department of Medical Microbiology, Faculty of Medicine, Bursa Uludağ University, Bursa 16059, Türkiye; harunagca@uludag.edu.tr

**Keywords:** low-level viremia, HIV, antiretroviral therapy, risk factors, CD4+ T-cell count, metabolic comorbidity

## Abstract

Background: Durable virological suppression is achieved in the majority of people living with HIV (PLWH) receiving contemporary antiretroviral therapy (ART). However, a subset of patients experience persistent low-level viremia (LLV), the clinical relevance and underlying determinants of which remain incompletely understood. Methods: Adult PLWH followed between 1 January 2005 and 31 December 2025 were retrospectively evaluated. LLV was defined as detectable HIV-1 RNA < 200 copies/mL on at least two consecutive measurements during follow-up in individuals receiving ART for at least 6 months. Patients with sustained virological suppression served as controls. Propensity score matching (1:1) was performed using variables associated with LLV in univariate analyses. Multivariable logistic regression analysis was applied to identify factors independently associated with LLV, and a *p* value < 0.05 was considered statistically significant. Results: Among 880 PLWH, 45 patients with LLV and 113 virologically suppressed controls were included. LLV was associated with lower baseline CD4+ T-cell counts, higher baseline HIV-1 RNA levels, delayed virological suppression at weeks 8 and 24, increased frequency of AIDS-defining illnesses and a higher prevalence of metabolic comorbidities, including hypertension, diabetes mellitus or dyslipidemia in univariate analysis. After propensity score matching, 32 patients remained in each group, with no clear association between low-level viremia and antiretroviral regimen class. Multivariable regression analysis showed baseline CD4+ T-cell count < 200 cells/µL, and the presence of ≥1 metabolic comorbidity (hypertension, diabetes mellitus or dyslipidemia) remained independently associated with LLV. Conclusions: Our findings suggest that LLV is associated with host-related factors rather than antiretroviral regimen failure. The coexistence of immunological impairment and metabolic comorbidities in patients with LLV underscores the importance of comprehensive clinical evaluation.

## 1. Introduction

Recent advances in combination antiretroviral therapy (ART) have enabled durable virological suppression in the vast majority of people living with HIV (PLWH), yet in routine clinical practice a subset of patients does not sustain plasma HIV-1 RNA levels below 50 copies/mL and instead exhibits persistent low-level detectable viremia, commonly referred to as low-level viremia (LLV) [1].

Low-level viremia may occur even among patients with good treatment adherence receiving contemporary ART regimens. Multiple mechanisms have been proposed to explain this phenomenon, including intermittent activation of viral reservoirs, pharmacokinetic and pharmacodynamic variability of antiretroviral drugs, and host-related factors [2]. Recent studies have demonstrated that LLV is not a rare laboratory finding. A contemporary systematic review and meta-analysis reported a pooled LLV prevalence of approximately 13–15% among PLWH receiving ART and showed that the presence of LLV may be associated with an increased risk of subsequent virological failure [3].

Beyond its virological implications, LLV has also been associated with impaired immunological recovery. Longitudinal cohort studies have shown that patients with persistent or recurrent LLV experience smaller increases in CD4+ T-cell counts over time, suggesting a negative impact on immune reconstitution despite ongoing ART [4]. In addition, retrospective studies evaluating risk factors for virological failure among patients with LLV have highlighted the potential role of treatment-related characteristics and patient-specific factors in determining long-term outcomes [5].

Importantly, substantial heterogeneity exists across studies with regard to LLV definitions, follow-up durations, patient populations, and clinical endpoints. While some cohort analyses suggest that LLV may be transient and clinically insignificant, others report that persistent LLV is associated with adverse virological and clinical outcomes [6]. These conflicting findings contribute to ongoing uncertainty regarding the optimal clinical management of LLV and underscore the need for additional real-world data.

In this context, the evaluation of long-term follow-up data from local patient populations is crucial, both to address gaps in national evidence and to contribute to the international debate on the clinical relevance of LLV. Comparisons across different healthcare systems and patient profiles may further clarify the true clinical significance of low-level viremia. The aim of the present study was to evaluate the frequency and clinical characteristics of low-level viremia among adults living with HIV and to investigate the association between LLV, sustainability of virological response, and antiretroviral treatment regimens.

## 2. Materials and Methods

### 2.1. Study Design and Population

This retrospective cohort study included adult PLWH (≥18 years) who were regularly followed at the Department of Infectious Diseases and Clinical Microbiology, Bursa Uludag University Faculty of Medicine, between 1 January 2005 and 31 December 2025. Patients with available longitudinal HIV-1 RNA and CD4+ T-cell count data after ART initiation were eligible for inclusion.

### 2.2. Definitions

LLV was defined as detectable HIV-1 RNA < 200 copies/mL on at least two consecutive measurements in individuals receiving ART for at least 6 months, and ART regimen modifications solely due to LLV were not routinely performed during follow-up, consistent with current guidelines [1,7,8]. Viral blips were defined as isolated detectable HIV-1 RNA measurements following virological suppression, with subsequent return to undetectable levels. Patients who achieved and maintained sustained virological suppression throughout follow-up were classified as the control group.

According to the European AIDS Clinical Society (EACS) guidelines, hypertension was defined as systolic blood pressure ≥ 140 mmHg and/or diastolic blood pressure ≥ 90 mmHg or current use of antihypertensive medication, dyslipidemia as the presence of abnormal lipid parameters including elevated total cholesterol, LDL cholesterol, triglycerides and/or reduced HDL cholesterol or use of lipid-lowering therapy, and diabetes mellitus as a documented diagnosis of type 2 diabetes, use of antidiabetic medication, or elevated glycemic parameters consistent with guideline thresholds [7].

Cytomegalovirus (CMV) infection was defined as the detection of CMV DNA by quantitative polymerase chain reaction (PCR) in any body fluid samples, including blood/serum, cerebrospinal fluid, and bronchoalveolar lavage specimens, or tissue specimens during the follow-up period [9]. AIDS-defining illnesses were classified according to the 1993 CDC revised classification system for HIV infection and AIDS surveillance definition [10].

### 2.3. Data Collection

Demographic characteristics, HIV transmission routes, lifestyle factors, comorbidities (including dyslipidemia, hypertension, or diabetes mellitus), opportunistic infections, antiretroviral treatment regimens, and concomitant medication use were extracted from electronic medical records. Baseline laboratory parameters at ART initiation included HIV-1 RNA level, CD4+ and CD8+ T-cell counts, CD4/CD8 ratio, and metabolic markers. Eligibility criteria were defined to ensure a homogeneous study population and to allow an accurate comparison between individuals with low-level viremia and virologically suppressed controls.

Inclusion criteria

- Age ≥ 18 years;
- Documented diagnosis of HIV infection;
- Receipt of antiretroviral therapy for at least 6 months;
- Availability of at least two HIV-1 RNA measurements during the study period (between 1 January 2005 and 31 December 2025);
- Classification into one of the following groups:
- LLV group: receipt of continuous antiretroviral therapy for at least 6 months and at least two consecutive HIV-1 RNA measurements with detectable viral load < 200 copies/mL;
- Control group: sustained virological suppression with HIV-1 RNA persistently below the limit of detection during the same period.

Exclusion criteria

- Unknown dates of ART initiation or discontinuation.
- Patients with clearly documented irregular ART use or treatment interruption/discontinuation during follow-up were excluded. Among excluded patients, the shortest documented interruption duration identified in the medical records was 1 month; therefore, interruption/discontinuation ≥1 month was used as the operational exclusion criterion.
- Insufficient follow-up data for virological evaluation (less than 6 months of follow-up).

### 2.4. Statistical Analysis

Continuous variables were expressed as mean ± standard deviation or median (interquartile range) and compared using Student’s *t*-test or the Mann–Whitney U test, as appropriate. *p* values presented in Table 1 represent results of univariate binary logistic regression analyses and were used for descriptive comparison between groups. Fisher’s exact test was additionally performed for variables with low expected frequencies or zero cell counts.

To minimize baseline differences between groups, propensity score matching (PSM) was performed using variables that were significant in univariate analyses (*p* < 0.05). Matching was conducted using a 1:1 nearest-neighbor method without replacement and a caliper width of 0.3 standard deviations of the logit of the propensity score.

Multivariable logistic regression analysis was used to identify factors independently associated with LLV. To avoid multicollinearity, variables representing overlapping clinical constructs were not simultaneously included. Variables included in the multivariable model were selected based on clinical relevance, statistical significance in univariate analyses, and the need to avoid model overfitting considering the limited sample size. Model fit was assessed using the chi-square test and Nagelkerke R^2^.

### 2.5. Ethical Considerations

Ethics committee approval was obtained from Bursa Uludağ University Health Research Ethics Committee on 4 February 2026 with a number of 2026/71/3-15. The study was conducted in accordance with the Declaration of Helsinki.

## 3. Results

Among 880 PLWH under follow-up, 45 patients with LLV and 113 virologically suppressed controls met the inclusion criteria. Baseline demographic and clinical characteristics are summarized in Table 1.

**Table 1:** Demographic characteristics, transmission routes, lifestyle factors, comorbidities, opportunistic infections, and ART-related variables.

Variable	LLV (*n* = 45)	Control (*n* = 113)	*p* Value *
Age, years (mean ± SD)	43.31 ± 1.92	40.38 ± 1.03	0.205
Male sex	45/45 (100%)	100/113 (89%)	0.999
Men who have sex with men (MSM)	10/45 (22%)	21/113 (18%)	0.766
Heterosexual	17/45 (38%)	37/113 (33%)	0.677
Unknown transmission	18/45 (40%)	55/113 (49%)	0.418
Prisoner	1/45 (2%)	3/113 (3%)	0.876
Diabetes mellitus	8/45 (18%)	5/113 (4%)	0.010
Hypertension	8/45 (18%)	7/113 (6%)	0.032
Dyslipidemia	7/45 (16%)	5/113 (4%)	0.025
Chronic obstructive pulmonary disease	1/45 (2%)	4/113 (4%)	0.672
Coronary artery disease	4/45 (9%)	4/113 (4%)	0.181
Chronic hepatitis B	1/45 (2%)	2/113 (2%)	0.851
Chronic hepatitis C	0/45 (0%)	2/113 (2%)	1.000 **
Smoking	23/42 (55%)	78/109 (72%)	0.052
Alcohol use	18/42 (43%)	47/109 (43%)	0.977
Intravenous drug use/substance	2/45 (4%)	5/113 (4%)	0.847
Syphilis	14/45 (31%)	33/113 (29%)	0.813
Tuberculosis	3/45 (7%)	1/113 (1%)	0.070 **
Cerebral toxoplasmosis	3/45 (7%)	0/113 (0%)	0.022 **
CMV infection	7/45 (16%)	3/113 (3%)	0.008
CMV retinitis	2/45 (4%)	0/113 (0%)	0.080 **
Gastroenteritis	3/45 (7%)	5/113 (4%)	0.564
*Pneumocystis jirovecii* pneumonia	4/45 (9%)	5/113 (4%)	0.284
HSV infection	1/45 (2%)	3/113 (3%)	0.876
Lymphoma	1/45 (2%)	0/113 (0%)	0.285 **
Kaposi sarcoma	2/45 (4%)	0/113 (0%)	0.080 **
ART change due to drug–drug interaction	6/45 (13%)	6/113 (5%)	0.096
Concomitant drug/supplement use affecting ART	5/45 (11%)	0/113 (0%)	0.002 **
Viral suppression at week 8	17/40 (43%)	60/88 (68%)	0.006
Viral suppression at week 24	23/39 (59%)	85/104 (82%)	0.002
Detected ART resistance	0/10 (0%)	7/54 (13%)	0.193 **

* *p* values were derived from univariate binary logistic regression analyses. ** Fisher’s exact test *p* values were additionally performed for variables with low expected frequencies or zero cell counts.

Cerebral toxoplasmosis was observed only in the LLV group and was significantly more frequent compared with controls based on Fisher’s exact test analysis (7% vs. 0%, *p* = 0.022). Tuberculosis (7% vs. 1%, *p* = 0.070), CMV retinitis (4% vs. 0%, *p* = 0.080), and Kaposi sarcoma (4% vs. 0%, *p* = 0.080) were also more frequently observed in the LLV group. In addition, CMV infection was significantly more common in the LLV group compared with the control group (16% vs. 3%, *p* = 0.008) (Table 1). Patients with LLV had a significantly higher prevalence of diabetes mellitus, hypertension, and dyslipidemia compared with controls. Viral suppression rates at weeks 8 and 24 after ART initiation were significantly lower in the LLV group. Baseline laboratory analyses demonstrated higher HIV-1 RNA levels and significantly lower CD4+ T-cell counts among patients with LLV (Table 2). Sequential HIV-1 RNA measurements of the 45 participants with LLV were reviewed to better characterize longitudinal virological patterns during follow-up. The median most recently measured HIV-1 RNA level was 50 copies/mL (range: 18–171 copies/mL), while the median immediately preceding HIV-1 RNA level was 51 copies/mL (range: 19–182 copies/mL). No participant had HIV-1 RNA ≥ 200 copies/mL in either the most recent or immediately preceding sequential measurements.

After PSM, 32 patients remained in each group with comparable baseline antiretroviral regimen characteristics (Table S1). The majority of patients in both groups were receiving INSTI-based regimens, and ART regimen class was not independently associated with LLV (Table 3).

In multivariable logistic regression analysis, baseline CD4+ T-cell count < 200 cells/µL and the presence of at least one metabolic comorbidity (hypertension, diabetes mellitus, or dyslipidemia) were independently associated with LLV development (Table 4). The presence of at least one AIDS-defining illness, including tuberculosis, CMV infection/CMV retinitis, toxoplasmosis, *Pneumocystis jirovecii* pneumonia, Kaposi sarcoma, and lymphoma, was significantly more frequent in the LLV group compared with the control group in univariate analysis (26.7% vs. 5.3%, *p* < 0.001). However, this association did not remain statistically significant in multivariable analysis (*p* = 0.191).

## 4. Discussion

LLV among PLWH receiving ART represents a heterogeneous clinical phenomenon, the clinical relevance of which has been increasingly discussed in the era of contemporary high-genetic-barrier regimens. In the present study, LLV was independently associated with advanced immunosuppression and a greater burden of metabolic comorbidities, including hypertension, diabetes mellitus, and dyslipidemia, whereas no clear association was observed with antiretroviral regimen class. These findings are in line with emerging evidence suggesting that LLV may be more closely related to host-related factors than to intrinsic antiretroviral treatment failure [11].

From a biological standpoint, LLV is thought to reflect intermittent viral transcription from long-lived infected cell clones rather than sustained rounds of productive viral replication. In a mechanistic study, Bachmann et al. demonstrated that viral blips and persistent LLV were associated with slower decay of the HIV reservoir despite suppressive ART, suggesting ongoing low-grade viral activity even under effective treatment [12]. This reservoir-driven model provides a plausible biological explanation for our findings, in which LLV coexists with apparent virological control but is accompanied by suboptimal immunological recovery.

CMV-specific CD4+ T-cells constitute a substantial proportion of the total CD4+ T-cell pool in seropositive individuals and expand early after ART initiation [13]. Persistent CMV antigenic stimulation may promote clonal expansion of differentiated effector-memory CD4+ T-cells, contributing to immune activation and contraction of the naïve CD4+ T-cell compartment. Given that gut-associated lymphoid tissue (GALT) represents a major reservoir for both CMV persistence and HIV latency, chronic CMV-driven immune activation within mucosal compartments may facilitate localized HIV transcription or expansion of HIV-infected CD4+ T-cell clones. In this context, CMV may contribute to LLV through antigen-driven clonal dynamics rather than overt viral reactivation. Although CMV infection was significantly associated with LLV in univariate analysis, this association did not persist after multivariate adjustment, possibly reflecting limited statistical power due to the relatively small number of CMV events in our study.

Current expert recommendations indicate that confirmed HIV-1 RNA levels below 200 copies/mL do not meet the definition of virological failure and do not warrant routine modification of ART in adherent patients. Instead, individualized clinical assessment and close virological monitoring are emphasized [14], a position that is consistently supported by international treatment guidelines [7,8]. Our data align with this approach, as LLV in our cohort was not primarily associated with ART regimen characteristics.

A potentially bidirectional relationship may exist between LLV and metabolic comorbidities, as persistent low-grade viral replication may promote chronic inflammation and metabolic dysfunction, while pre-existing metabolic disorders may also impair viral control, suggesting a self-perpetuating cycle. Beyond virological considerations, the association between LLV and metabolic comorbidities observed in our study is particularly noteworthy. In a large retrospective cohort study by Wang et al., including 848 PLWH followed for a median of 3.9 years, 31.3% of participants developed metabolic syndrome. The incidence rates of metabolic syndrome were markedly higher among individuals with LLV, reaching 160.4 and 136.6 cases per 1000 person-years in the LLV 201–500 and LLV 51–200 copies/mL groups, respectively, compared with 83.0 cases per 1000 person-years in patients with fully suppressed viremia [15]. In time-dependent Cox regression analyses, LLV remained an independent predictor of metabolic syndrome irrespective of ART regimen [15]. In our cohort, although metabolic syndrome incidence was not evaluated longitudinally, the higher prevalence of metabolic comorbidities among individuals with LLV is concordant with these findings and suggests a shared metabolic vulnerability.

Emerging evidence further indicates that LLV may contribute to the development of specific metabolic diseases. In a large longitudinal cohort study from China, Tao et al. reported that LLV was associated with a significantly increased risk of incident diabetes mellitus [16]. After propensity score matching, LLV was associated with a 27% higher risk of diabetes (adjusted hazard ratio [aHR] 1.27; *p* = 0.012) [16]. Notably, in individuals aged 35–49 years, the risk was substantially higher, with an aHR of 2.03 for persistent LLV and 1.36 for viral blips compared with virologically suppressed patients [16]. In our study, the higher frequency of diabetes mellitus among patients with LLV supports the clinical relevance of these observations and underscores the need for integrated metabolic monitoring in this population.

The relationship between low-level viremia and cardiovascular risk appears to be heterogeneous across different populations and study settings. In a cross-sectional study by Botha-Le Roux et al., 208 South African people living with HIV receiving ART were evaluated, among whom 46% had LLV (50–999 copies/mL) [17]. In that cohort, no significant differences were observed between individuals with LLV and those with virological suppression with respect to arterial stiffness, carotid intima–media thickness, or a broad range of cardiovascular biomarkers [17]. These findings suggest that the cardiovascular implications of LLV may depend on population characteristics, baseline cardiovascular risk, and study design. In our cohort, although no difference was observed in overt coronary artery disease, the higher prevalence of cardiometabolic comorbidities—particularly hypertension—among patients with LLV may reflect earlier or subclinical cardiovascular risk profiles, potentially influenced by differences in age distribution, comorbidity burden, and duration of HIV infection and ART exposure.

At the population level, a recent systematic review and meta-analysis by Zhang et al. reported that low-level viremia affects approximately 10–15% of treated people living with HIV and is more frequently observed among individuals with lower CD4+ T-cell counts and host-related risk factors, rather than reflecting antiretroviral treatment failure per se [18]. Complementing these findings, longitudinal data from the Swiss HIV Cohort Study reported by Lanz et al. demonstrated that the presence of LLV was associated with an increased risk of subsequent virological failure, indicating that LLV cannot be considered uniformly benign across all patient populations [19]. Importantly, our findings add clinical context to these observations by suggesting that this increased risk may be more pronounced among individuals with advanced immunosuppression and a higher burden of metabolic comorbidities. In this respect, LLV may function as a marker of host-related vulnerability, identifying patients who may benefit from closer virological monitoring rather than immediate modifications to antiretroviral therapy.

National data from Türkiye remain limited. Studies by Akbulut et al. have shown that LLV and unsuppressed viremia are relatively common in routine clinical practice and are influenced by host-related factors, including immunological status and comorbidity burden [20,21]. Our findings are consistent with these observations and extend them by demonstrating that LLV below conventional virological failure thresholds is associated with clinically meaningful metabolic and immunological characteristics in a real-world setting.

Previous studies have shown that LLV may be associated with increased clinical progression and a higher burden of both AIDS-defining and non-AIDS-defining comorbidities among PLWH [22,23,24]. Persistent low-level viremia may contribute to ongoing immune activation and impaired immune recovery, thereby increasing susceptibility to opportunistic infections and AIDS-defining conditions. In our cohort, the presence of at least one AIDS-defining illness, including tuberculosis, CMV infection/CMV retinitis, toxoplasmosis, *Pneumocystis jirovecii* pneumonia, Kaposi sarcoma, and lymphoma, was significantly more frequent in the LLV group in univariate analysis. These observations may reflect the complex interaction between persistent low-level viral replication, immune dysregulation, and advanced HIV disease. However, the loss of statistical significance after multivariable adjustment suggests that baseline immunological status and other clinical confounders likely contribute to the observed association. In addition, the relatively limited number of AIDS-defining events in our cohort may have reduced the statistical power to detect an independent association in multivariable analysis.

The strengths of this study include detailed clinical characterization and the simultaneous evaluation of virological, immunological, and metabolic parameters. Limitations include the retrospective single-center design, limited sample size, absence of molecular resistance and viral reservoir analyses, and lack of longitudinal assessment of non-AIDS clinical outcomes. No resistance mutations were detected among tested LLV participants; however, systematic resistance analyses were not available for all individuals because of the retrospective design, and emerging resistant viral variants cannot be completely excluded. Although propensity score matching was applied, residual imbalance in selected covariates persisted as reflected by standardized mean differences, and residual confounding cannot be completely excluded. In addition, body mass index data were not consistently available due to the retrospective design of the study. Since INSTI-based regimens may contribute to weight gain and metabolic alterations, this should be considered when interpreting metabolic comorbidities in patients with LLV. Previous studies have shown that advanced immunosuppression and lower CD4+ T-cell counts may contribute to persistent immune activation, chronic inflammation, and increased metabolic comorbidity burden among PLWH [25,26]. Therefore, the observed association between LLV and metabolic comorbidities in our cohort may partly reflect differences in disease stage and treatment exposure rather than a direct causal relationship.

## 5. Conclusions

Low-level viremia among PLWH receiving ART is independently associated with advanced immunosuppression and metabolic comorbidities, including hypertension, diabetes mellitus or dyslipidemia. These findings support the interpretation of LLV as a marker of host-related vulnerability rather than antiretroviral regimen failure. Individualized follow-up strategies focusing on immune recovery, metabolic health, and close virological monitoring are essential to optimize long-term outcomes.

## Figures and Tables

**Table 2:** Laboratory characteristics at ART initiation.

Variable	LLV (*n* = 45)	Control (*n* = 113)	*p* Value *
HIV-1 RNA (log10 copies/mL)	5.22 ± 0.16	4.82 ± 0.09	0.029
CD4 count (cells/µL)	247 ± 29	480 ± 24	<0.001
CD4/CD8 ratio	0.42 ± 0.08	0.59 ± 0.05	0.096
HbA1c (%)	5.8 ± 0.16 (*n* = 38)	5.4 ± 0.04 (*n* = 94)	0.010
LDL (mg/dL)	94.47 ± 5.2	103.7 ± 2.04	0.122
HDL (mg/dL)	36.31 ± 1.79	41.67 ± 1.62	0.058
Total cholesterol (mg/dL)	152.06 ± 6.19	169.55 ± 3.63	0.015
Triglycerides (mg/dL)	137.31 ± 12.59	136.88 ± 7.09	0.975
Total cholesterol/HDL	4.35 ± 0.17	5.03 ± 0.44	0.361

* Student’s *t*-test or Mann–Whitney U test was used, as appropriate.

**Table 3:** Antiretroviral treatment characteristics after propensity score matching.

Variable	LLV (*n* = 32)	Control (*n* = 32)	*p* Value *
INSTI-based regimen at ART initiation	31/32 (97%)	28/32 (88%)	0.195
PI-based regimen at ART initiation	0/32 (0%)	2/32 (6%)	0.492 **
NNRTI-based regimen at ART initiation	1/32 (3%)	2/32 (6%)	0.562
INSTI-based current regimen	31/32 (97%)	31/32 (97%)	1.000
PI-based current regimen	1/32 (3%)	1/32 (3%)	1.000
NNRTI-based current regimen	0/32 (0%)	1/32 (3%)	1.000 **

* *p* values were derived from univariate binary logistic regression analyses. ** Fisher’s exact test *p* values were additionally performed for variables with low expected frequencies or zero cell counts.

**Table 4:** Multivariable logistic regression analysis for LLV development.

Variable	Odds Ratio	95% CI	*p* Value
Baseline CD4+ T-cell count < 200 cells/µL	3.472	1.275–9.451	0.015
Having at least one metabolic comorbidity *	3.615	1.214–10.179	0.015
No viral suppression at week 8	1.718	0.663–4.451	0.265
Having at least one AIDS-defining illness **	2.667	0.614–11.586	0.191
Baseline HIV-1 RNA ≥ 10^5^ copies/mL	1.085	0.421–2.793	0.866

* Hypertension, diabetes mellitus, or dyslipidemia. ** Tuberculosis, CMV infection/CMV retinitis, toxoplasmosis, *Pneumocystis jirovecii* pneumonia, Kaposi sarcoma, lymphoma.

## Data Availability

The data supporting the findings of this study are available from the corresponding author upon reasonable request. Data are not publicly available due to privacy and ethical restrictions.

## Supplementary Materials

The following supporting information can be downloaded at: https://www.mdpi.com/article/10.3390/v18060611/s1, Table S1: Covariate balance after propensity score matching.

## Abbreviations

The following abbreviations are used in this manuscript:

LLV	Low-level viremia
ART	Antiretroviral therapy
PLWH	People living with HIV
CMV	Cytomegalovirus
HSV	Herpes simplex virus
PI	Protease inhibitors
INSTI	Integrase inhibitors
NNRTI	Non-nucleoside reverse transcriptase inhibitors
