# Supplementary material for: Low-Level Viremia in People Living with HIV: A Retrospective Cohort Study
Source: Viruses. 2026 May 27;18(6):611. doi: 10.3390/v18060611 (PMC13307737; doi:10.3390/v18060611)
Supplement: Supplementary file 1 [file viruses-18-00611-s001.zip › viruses-4324989-supplementary.pdf]

Table S1. Covariate balance after propensity score matching*

Variable	LLV (n=32)	Control (n=32)	SMD
**Age, years**	41.28	43.22	-0.169
**Male sex**	1.00	0.84	0.582
**HIV-1 RNA at diagnosis (log10 copies/mL)**	4.99	5.38	-0.398
**CD4 count at diagnosis (cells/μL)**	292.70	291.25	0.007
**HbA1c (%)**	5.73	5.45	0.427
**Total cholesterol (mg/dL)**	158.16	163.75	-0.132
**LDL cholesterol (mg/dL)**	97.45	103.36	-0.165
**HDL cholesterol (mg/dL)**	38.59	40.81	-0.144
**Triglycerides (mg/dL)**	135.50	131.38	0.054
**Hypertension**	0.12	0.09	0.100
**Smoking**	0.43	0.23	0.441
**CMV infection**	0.12	0.09	0.100
**Viral suppression at week 8**	0.43	0.46	-0.057
**Viral suppression at week 24**	0.36	0.28	0.175

*Propensity score matching was performed using variables that were statistically significant in univariate analyses ($p<0.05$). To improve interpretability of balance diagnostics, continuous metabolic parameters (HbA1c and lipid fractions) were used instead of categorical metabolic comorbidities. Standardized mean differences (SMDs) are shown for key baseline covariates. An absolute SMD <0.10 indicates adequate balance. Residual imbalance for selected covariates persisted after matching, reflecting the intentional restriction of the propensity score model to variables significant in univariate analyses.
